# Belt and road initiative as a catalyst of infrastructure development: Assessment of resident’s perception and attitude towards China-Pakistan Economic Corridor

**DOI:** 10.1371/journal.pone.0271243

**Published:** 2022-07-20

**Authors:** Shahid Mahmood, Ghaffar Ali, Rashid Menhas, Muazzam Sabir

**Affiliations:** 1 Government Associate College (Commerce), Shorkot City, Punjab, Pakistan; 2 College of Management, Shenzhen University, Shenzhen, Guangdong, China; 3 Research Center of Sport Social Sciences, School of Physical Education and Sports, Soochow University China, Suzhou, Jiangsu, China; 4 College of Agriculture, University of Sargodha, Sargodha, Pakistan; Al Mansour University College-Baghdad-Iraq, IRAQ

## Abstract

The Chinese Government commenced the One Belt One Road (OBOR) project in 2013 for the multidimensional development to restore the historical Silk Road paradigms. The Economic Corridor provides networks and connections based on two geographical territories. The China Pakistan Economic Corridor (CPEC) is one of them, a chunk of the Silk Road Economic Belt’s economic corridors. CPEC would be an economic game-changer for Pakistan. It will generate businesses, minimize poverty, generate employment opportunities, and improve local communities’ health and education. However, it is more lucrative for the Chinese economy because CPEC is a safe, cheapest, and shortest route for importing energy rather than other routes. The present study investigates the social, infrastructural, and tourism effects of CPEC through the mediation role of rural development and knowledge sharing, including income, employment, business, land-use change variables, and CPEC adoptability for a local community in Pakistan. This study is based on four different regions of Gilgit Baltistan, Khyber Pakhtunkhwa, Punjab, and Sindh in Pakistan. The study incorporates the collected data from the respondents living on the CPEC routes via face-to-face interviews from citizens (cities, villages, and towns). Besides, the study applied univariate and Structural Equation Model techniques to draw the results. The study results reveal that CPEC plays a significant role in Pakistan’s socio-economic and rural development. This mega project’s expectations might bring positive changes in infrastructure development, energy sector, and social development projects in Pakistan. The Results also indicated that CPEC would link rural areas to urban areas, which would offer development opportunities for rural regions to achieve sustainable development.

## 1. Introduction

One Belt One Road (OBOR) is one of the projects commenced in 2013 by the Chinese Government for the development of the economy to restore the historical Silk Road paradigms. The purpose of the restoration of Silk Road trade routes, such as Europe, Africa, and Asia via the development of infrastructure, optical fiber system, airports, railways, seaports, and roads network, as well as to empower the countries in the field of industry, energy power, and agriculture which are participating in this initiative [1,2]. Maritime Silk Road (by developing the ports), Silk Road Economic Belt (by connecting the geographical territories, having six economic corridors), Maritime Silk Road (by expanding the ports), Silk Road Economic Belt (by clicking the geographical regions, having six economic corridors), and Digital Silk Road (by promoting the domestic industry to participate in the global economy) are the significant mechanisms of Belt and Road initiative which will connect China with other countries [3,4]. Economic Corridor (EC) is a link of network or connection between two geographical regions, and the CPEC is one of them, which is a part of economic corridors of the Silk Road Economic Belt [5,6]. Free economic zones, the infrastructure of transportation, production of energy, and special economic zones are the part of CPEC that will encounter the interests and cooperation between Pakistan and China and significantly affect bordering countries. To support local connectivity, the Chinese Government focused on uplifting the economy, increase the export, create more job opportunities, increasing investment-related opportunities, and increasing the connections between other countries [7,8]. China is aimed to invest over 60 billion USD on mega projects in Pakistan concerned to CPEC by connecting the Gwadar port to Khunjerab pass, China-Pakistan border over pipelines, roads network, and railways with the distance of 3000 km, which will be helpful enhancing the trade activities all over Pakistan [9].

The development of transport and road infrastructure will impact the local community’s positivity, and business activities will also be increased. Development in CPEC will also affect residents by increasing tourism, transportation, new business, and additional economic benefits in the states [9–11]. CPEC is supposed to benefit the people living in local communities through infrastructural changes, like road transport infrastructure and access to big cities, facilitating health, job opportunities, education, living standards, technology availability, and other facilities [12]. Another notable contribution to the transportation system under the umbrella of CPEC is the High-speed railway (HSR). HSR has gained popularity as a significant mode of transportation for the development of local communities. Zhou [13] investigated the role of HSR construction in urban land expansion. They found that due to HSR, land demand would increase to construct new routes and stations. Thus, urban land would be extended.

Previous studies such as [4,14] suggest that the tourism business environment may significantly impact the region by developing transportation and road infrastructure development. It may be assumed that it could support tourism and beneficial for the local community. Although few studies examined the CPEC development for local communities’ perceptive in terms of investment opportunities, education, health, and employment, they need to be reviewed and considered the community-based benefits for societal development [12,15]. This study is aimed to investigate the China-Pakistan Economic Corridor as a Catalyst of Infrastructure Development: Assessment of resident’s perception and attitude towards OBOR from Pakistan perspectives. To achieve the main objectives such as: to investigate the social, infrastructural and tourism effects of CPEC through the mediation role of rural development and knowledge sharing mainly; including income, employment, business, land-use change variables, infrastructure development, and to CPEC adoptability for a local community in Pakistan. Most of the studies emphasize the importance of CPEC with various aspects, challenges faced by implementing projects, social effects, and current developments in CPEC. Likewise, most of the studies are qualitative, while the public opinion which is in low number and not very effective to measure the impact of significant decisions. So, it is imperative to know the local residence opinion about the CPEC development project for business leaders, government officials, and officials because of the achievement of any development projects grounded on the local public opinion. Furthermore, the Pakistani community faces various problems, such as health issues, unemployment, and business de-valuation. Researchers emphasized local communities regarding different areas such as infrastructure growth, Pak-China relationship, and CPEC development to resolve all these issues. The present study is quite significant because it has tried to investigate the local communities’ views about the CPEC project performance. In view of the social exchange theory, this knowledge is imperative because the local people can prove to be riotous and may not support the CPEC mega project if they believe that the route is unfavorable to them. Since Pakistan is a stakeholder in the project, investigation of the local community’s attitude towards the CPEC route is crucial for not only project progression and completion but also for the feasibility and long-term effects of the project.

Moreover, this research adds to the existing theoretical and empirical body of literature on development initiatives and has crucial policy implications for CPEC authorities. To the best knowledge of the researcher, this is the first study of its kind that explored the CPEC development project from the perspective of local communities with respect to education and healthcare facilities, employment opportunities, Investment opportunities, and community-based advantages for social evolution and economic growth.

## 2. Literature review and theoretical background

### 2.1 CPEC infrastructure development

CPEC infrastructure development projects can create entrepreneurial ventures, and investment opportunities for residents, such as real estate investment, automobile workshops, and SMEs started from a low budget. Through the CPEC project, improving transportation, infrastructure, and road system will change people’s living standard, the attractiveness of the region, generate accessibility, increase residents’ status, and quality of life [16]. CPEC is a cluster of infrastructural projects that are in progress all over Pakistan. At the initial stage, the project’s worth was $46 billion, but its cost is $62 billion. This project connects the Gwadar port city in Pakistan to the Xinjiang province of China [12]. Furthermore, Menhas [4] discussed that China is investing in various infrastructure projects, including road infrastructure, ports, railways, industrial zones, telecommunication sector, health sectors, and Pakistan’s educational sectors under CPEC. The completion of these projects would be significant in uplifting Pakistan socially and economically. Guo [17] explored HSR will bring geographical and economic changes in both countries. It will be fruitful in many aspects including providing employment opportunities in local communities, boost the integration of transportation system, redistribution of economic resources, and generate investment multiplier. Following are the hypothesis of the study according to the objectives of the study.

**H1a.** Infrastructural development is positively associated with rural development.
**H1b**.There is a positive relationship between infrastructure development and knowledge sharing.

### 2.2 CPEC social development

CPEC is an economic game-changer for the country Pakistan. It will generate businesses, employment opportunities, create more job opportunities, control the electricity shortfall. It will also cause connectivity among urban and rural areas, an attraction for universal stakeholders, industrial revolutions, health promotion, and education for the local populations [18]. This flagship project creates thousands of new job opportunities for local communities, rural areas connected with big cities, the new educational centers should be open, health facilities also improve, adopted new technologies, and improve the social condition of Pakistan [12]. The CPEC will help combat poverty and groom society by providing several life facilities, including quality of health and education, primary markets, and banking with cheap rates [18]. The expected benefits of these unique projects are also to bring economic and social welfare and impact society by improving their living standard and way of thinking [4]. Based on the above discussion, we hypothesized:

**H2a.** There is a positive association between social development and rural development.
**H2b.** Social Development positively influences knowledge sharing.

### 2.3 CPEC tourism development

Towards tourism development, the local community’s satisfaction level related to positive tourism is an essential factor that inspirations the host community’s behavior [12] Kanwal et al. (2019a). Improved transport and road infrastructure provides a straightforward approach to tourism destinations and enhances economic activities that significantly impact the local communities’ living standard. With the development of this universal project, it is predicted that Pakistan’s northern regions, especially Khyber Pakhtunkhwa and Gilgit Baltistan, will convert the country’s tourism center. These areas comprise adorable sites, with resorts, lakes, and glaciers [19] (Mamirkulova et al., 2020). Several researchers have suggested that transport and road infrastructure play a vital role in increasing tourism activities and enhancing new tourism sites in the local areas [20,21]. Previous literature has shown a significant relationship among improved road and transport structure and tourism [22,23]. Consequently, we hypothesized:

**H3a:** Tourism Development positively influences rural development.
**H3b:** Tourism Development positively associated with knowledge sharing.

### 2.4 Mediating effect of CPEC rural development

Because of limited financial sources, entrepreneurial intention, and fewer market demands, rural areas’ residents cannot start their businesses, and the CPEC project will enhance this opportunity, rural individuals. In this way, poverty will decrease in rural and urban areas and significantly impact economic growth. Thus, the projects that have been started under CPEC consider the importance of better infrastructure for ordinary people residing in rural areas to make their lives better in every aspect [24–26]. CPEC can connect Pakistani rural areas with big cities (Karachi, Lahore, Islamabad, Faisalabad, and Balochistan), where peoples quickly move from one city to another and get batter education, access big markets, get batter health facilities and also start your own small level business Islamabad (2017) [27]. Likewise, several studies also argued that after the initiation of projects under CPEC, local communities have seen and perceived an improvement in infrastructure, employment opportunities, and health facilities, particularly in rural areas via CPEC routes that connect Pakistan’s rural and urban areas [15,28]. So the proposed hypothesis is as under:

**H4:** Rural Development positively influence CPEC adoptability.
**H5a:** Rural development play a positive mediating role toward Infrastructure development and CPEC adoptability.
**H5b:** Rural development mediates the positive relationship between social development and CPEC adoptability.
**H5c:** Rural development mediate the positive relationship among tourism development and CPEC adoptability

### 2.5 Mediating effect of knowledge sharing

China is focusing on technology and knowledge transfer under CPEC to improve the socio-economic structure. During the last years, several researchers have explored the impact of CPEC on knowledge sharing, especially in Pakistan’s local communities. Nowadays, knowledge sharing is the most commonly discussed term during the management of knowledge. According to [29], knowledge-seeking is frequently arranged in a particular setting, cultural and local societies. Under this setting, the way of learning is made, shared, and utilized. In the knowledge transfer system, it is essential that knowledge sharing must occur [30]. Similarly, sources of knowledge and transferring information are imperative to achieve developed local communities, especially in introducing new production techniques. Thus, knowledge sharing is vital to attain desired goals regarding underdeveloped communities [31,32]. Following are the hypothesis of the study according to the above discussions.

**H6:** Knowledge sharing is positively associated with CPEC adoptability.
**H7a:** Sharing of knowledge mediates the positive relationship between Infrastructure development and CPEC adoptability.
**H7b:** Knowledge sharing mediates the positive association toward social development and CPEC adoptability.
**H7c:** Sharing of knowledge mediates the direct relationship between tourism development and CPEC adoptability.

### 2.6 CPEC adoptability

**Fig 1:**
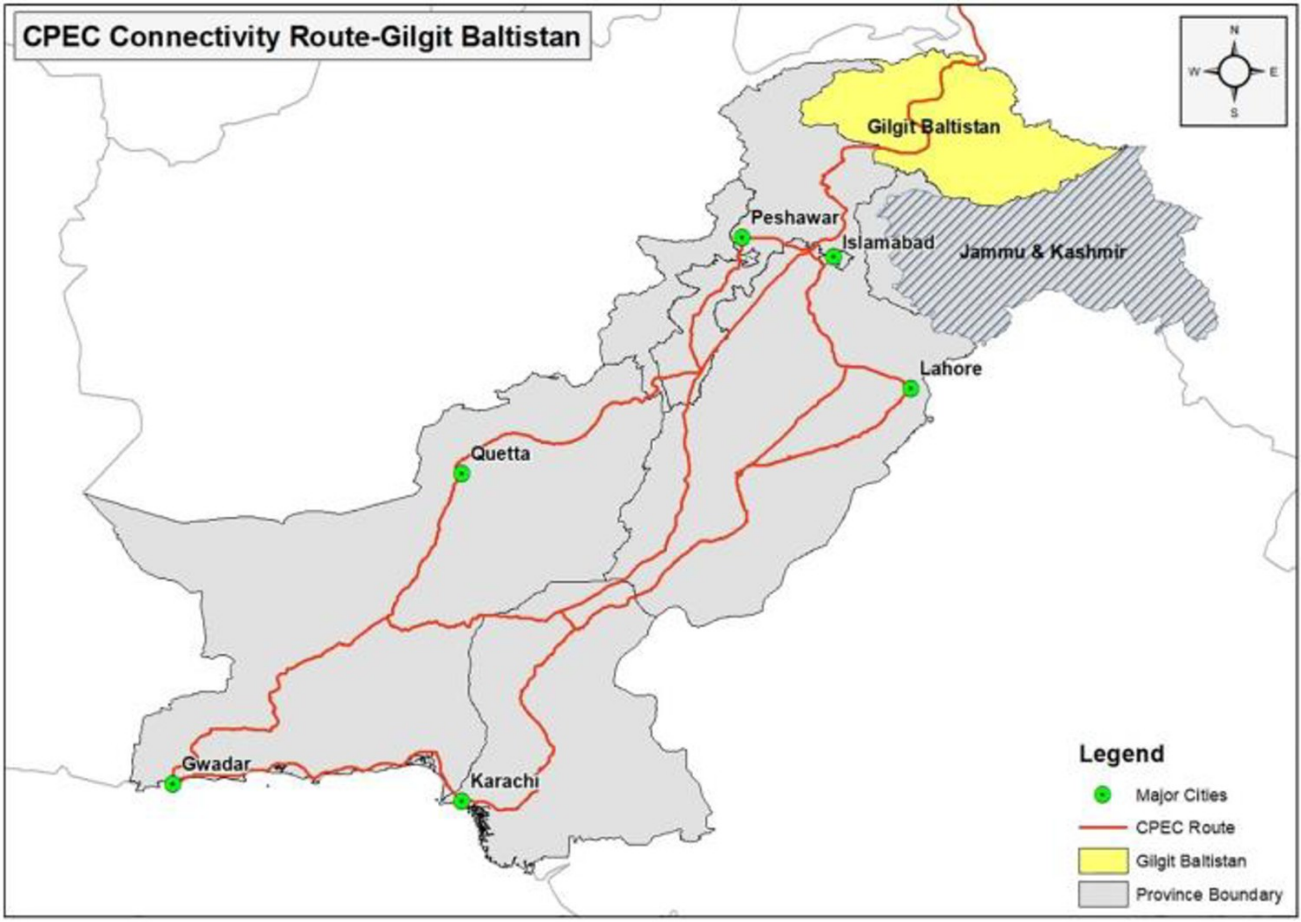
CPEC map silk road route in Pakistan from China. source: [12].

Pakistan’s geographical location makes it ideal because it links the Middle East, South Asia, Central Asia, and China. Due to its perfect position, Pakistan is considered the middle point for any economic activity or business trade. No doubt, it is expected that CPEC will bring many advantages like economic development opportunities in both countries, especially in Pakistan, because of its abundant natural and human resources [33]. Presently, CPEC project is under development in Pakistan, such as energy projects, Gwadar port development, road infrastructure, special economic zones, Lahore Mass transit, and fiber optics, etc. however, railway, industrial economic zones development, international Gwadar airport will be implemented shortly [6,34]. Map of CPEC is show in Fig 1. It is essential to recognize residents’ perception about the CPEC development project for policymakers, government officials, and business leaders what kind of parameters need to adopt, which will change their attitude during CPEC adoptability [12,35].

### 2.7 Social exchange theory

**Fig 2:**
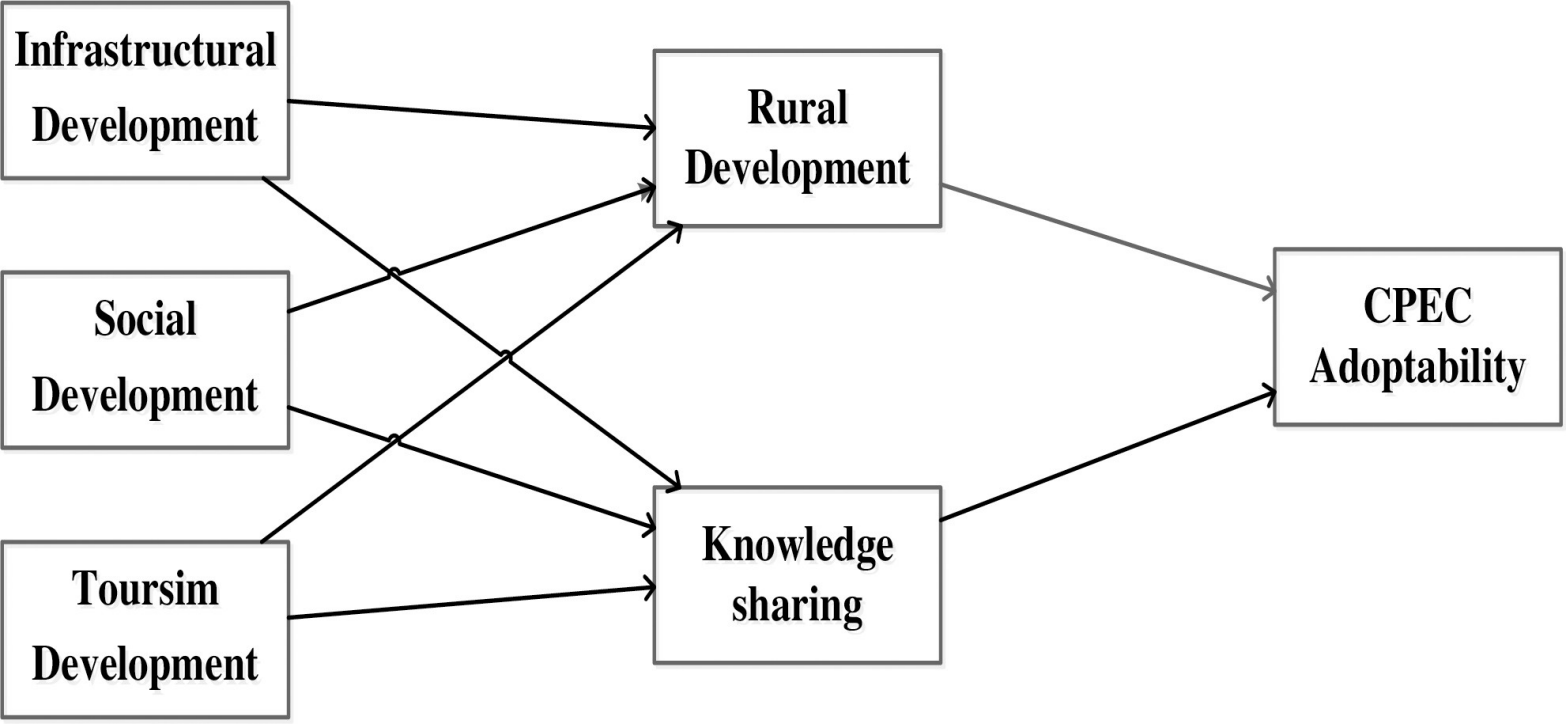
The proposed conceptual framework and hypothesis relationship of the study.

Social exchange theory (SET) implies assets among groups and individuals with mutual exchange possibilities, influencing local communities’ attitudes to supporting tourism, infrastructure, and transport development [11,36]. SET has been examined to consider the local community’s support, satisfaction, behavior, and attitude level suggested that residents evaluate the cost and benefits of perceiving development projects [37,38]. Mamirkulova [19] assessed that the local community’s behavior in the context of tourism development projects perceives personal interests and benefits. For instance, to CPEC development projects, social exchange theory suggests that host communities need to contribute to exchange perceiving; otherwise, the local community will negatively oppose the CPEC. SET also understands residents’ perceived effects (positive or negative), satisfaction, attitude, and personal benefits linked to CPEC [12,29]. Conceptual framework is shown in Fig 2. In short, if the local community will perceive unique benefits from CPEC routs, they will actively support and participate in its development. For that, support from the local community is essential form the success of CPEC development projects.

## 3. Research methodology

This research was carried out across Pakistan with the exception of Baluchistan and Azad Jammu and Kashmir (AJK). The data-gathering regions are certified to be on the CPEC highways or CPEC projects under construction. Hypotheses were tested by using PLS-SEM (Partial Least Squares Structural Equation Modelling) technique through SmartPLS V.3.2.9 package. SEM model was used to check the relation between different independent, mediation, and dependent variables. This study has utilized PLS-SEM (Partial Least Squares Structural Equation Modelling) technique for investigation, using a multivariate analysis which is implemented in SmartPLS. SmartPLS evaluates the measurement model’s psychometric qualities while also estimating the model’s parameters for its effective use in relation to time and situation [39]. It is an extensively used SEM technique that is variance-based, graphic, and prediction-oriented. SEM are complex methods of data analysis. In the social sciences, they allow for analyses that would not be possible using other methods. Even in cases where alternative methods of analyses are available, structural equation modeling may offer more meaningful and more valid results. On the other hand, more effort is necessary until the greater complexity pays off. Assumptions on the data may be higher, and the process of interpreting the results is more complex compared to other methods of data analysis.

### 3.1 Data collection procedure

A well-design questionnaire is used in this study to collect primary data. Both close and open-ended questions are used. The data were collected from the local community of Pakistan to observe the perceptive and opinion about CPEC in improving the quality of life, decreasing poverty, refining the education and health facilities, increasing employment opportunities, protecting the environment, and social interactions economic benefits, etc. A structured questionnaire was prepared according to the research’s nature based on a household survey from rural and urban areas. Interviews were conducted with Pakistan citizens (cities, towns, and villages). It is confirmed that all data collection areas are located on the CPEC routes or any other CPEC project is under construction, from all over the provinces excluding Baluchistan and Azad Jammu and Kashmir (AJK), such as Gilgit (Gilgit, Danyor, Jalalabad, Sultanabad, Gojal, Aliabad and Astor), Khyber Pakhtunkhwa (Abbottabad, Hawelian, Haripur, Mansehra, Sukki Kinari, Noshera, Risalpur, and Rashakei). The residents from Punjab province’s areas were interviewed, such as (Rawalpindi, Lahore, Faisalabad, Khanawal, Multan, and Bhawalpure) The participants were also selected from Sindh province of Pakistan (Sukkur, Rohri, Panu Aqil, and Hyderabad) connected to CPEC development projects [40]. The researcher spent four months in the field. All data were collected by himself, and no one provides any support (Financial, Transportation, living, or security). During the field survey, different participant statements are also noted down very carefully, and after the researcher rewrote that these minutes daily. Due to these reasons, the primary data’s reliabilities are further improved, and the researcher gets better results. The active participants were selected and well known about CPEC, well educated, and worked with the development projects and households’ land acquired. The questionnaires were distributed among 1050 respondents from the period of August to December 2019. However, the sample of 891 in the complete form was received among individuals, and the hard copy approach using non-probability random sampling method was used for the said purpose [41,42]. This study was established from previous literature, having six variables, such as CPEC adoptability, infrastructure development, tourism development, knowledge sharing, social and rural development linked to Pakistan’s perspectives. The variables, such as income, employment, business, land-use changes, infrastructure development, and CPEC adoptability were measured through the paradigms of [4,9,12,34,43–47], to observe the residents’ behavior shown in appendix 1. Questioners of this study have a five-point Likert scale, where 1 = strongly disagree and 5 = strongly agree. This study also encompassed demographic variables like gender, age, language, education level, and income level.

### 3.2 Pre-testing approach

Before collecting primary data into the field, it is better to check the questionnaire’s validity and accuracy. For this purpose, the pre-testing technique is used in this study. A total of 75 respondents is selected to check the validity of the data. After obtaining the respondents’ final responses, some valid modification was made in the final questionnaire.

## 4 Results of the study

### 4.1 Univariate analysis

Under this section, numerous statistical methods are used to examine the respondents’ demographics, likewise responded knowledge about the CPEC, societal benefits about CPEC projects, respondent’s suggestions for the effective operation of the CPEC projects, etc. Table 1 shows that a large majority (84.7%) of the participant were male, and only (15.3%) of the females are contributed to this survey. Moreover, (24.4%) of the respondents belong to the urban area and (75.6%) living background is a rural area because most of the CPEC projects are going to be established in rural areas in Pakistan that’s why most of the respondents also belong to the rural areas. This data also illustrates that approximately (75.1%) of the selected respondents were married, although (24.9%) were unmarried. The result also indicates that (13.5%) of the selected community whole family have under 30 years of education, (24.2%) had 30–40 years of education. However, (26.5%) of them were having 41–50 years of education, while (27.7%) having 51–60 years of education, and the remaining (18.3%) of them had 61–70 years of education. Data also demonstrate that (22.6%) of the respondents belonged to the farmer, an additional (19.4%) of them were government employee, further (16.3%) of them are laborers, (8.8%) are private employee, (24.2%) were businessman, another (7.7%) of the students and (1.0%) are a housewife. Moreover (16.6%) of the selected respondent’s total family income is under 50,000 PKR (Pakistani local currency), while (32.2%) of the income level is 50,001 to 100,000 PKR, (20.4%), fall under 100,001–150,000 this category and (9.9%) have 150,001–200,000, 200,001–250,000 and more than 250,001 respectively. Additionally, data reveal that (16.4%) of the respondents ages are under the age of 20 years, while (30.5%) of them had 20–30 years of age, (27.8%) had 31–40 years of age, and (16.0%) of the respondents had 41–50, and (9.2%) over 51 years of age accordingly. Likewise, (20.0%) of the respondent’s local language is Punjabi, while (11.4%) of them understand the Hinko language, 10.5, 10.2, 23.6,23.0 and (1.2%) of the selected community local language is Shina, Pashto, Sindhi, Saraiki, and Brushki respectively.

**Table 1:** Demographic detail of the participants.

	Frequency	Percentage
Female	136	15.3
Male	755	84.7
Urban Area	218	24.4
Rural Area	673	75.6
Married	669	75.1
Single	622	24.9
Under 30 years of education	120	13.5
30–40 years of education	216	24.2
41–50 years of education	242	26.5
51–60 years of education	163	27.2
61–70 years of education	150	18.3
Farmer	201	22.6
G. Employee	173	19.4
Labor	145	16.3
P. Employee	78	8.8
Businessman	216	24.2
Student	69	7.7
Housewife	9	1.0
Under 50,000 PKR	148	16.6
50,001–100,000 PKR	314	32.2
100,001–150,000 PKR	185	20.
150,001–200,0000 PKR	88	9.9
200,001–250,0000 PKR	57	6.4
Above 250,001 PKR	99	1.1
Under 20	146	16.4
20–30	272	30.5
31–40	248	27.8
41–50	143	16.0
Over 51	82	9.2
Punjabi	178	20.0
Hinko	102	11.4
Shina	94	10.5
Pashto	91	10.2
Sindhi	210	23.6
Saraiki	205	23.0
Brushki	11	1.2

Table 2 shows that in the selected area of the study, all the respondents knew about CPEC. According to Newspaper [48] in Pakistan, most of the community well know about CPEC. The Government of Pakistan also established an official website related to CPEC projects for getting more accurate information. Furthermore, (24.4%) of the selected respondents stated that they get CPEC projects related information over electronic media, (16.6%) get information through print media. In comparison (45.7%) of them get information through both sources, whereas (13.4%) of the respondents get information through other sources, i.e., relatives, friends, researchers, police, and military personals, etc. Results of the study also depict that (17.8%) of the respondents had thinking that CPEC is a road through which China and Pakistan are going to be connected, whereas roundabout (18.4%) of the selected respondents had believed that it is the most significant investment of China in Pakistan, whereas (17.7%) of them were perceived that this project is a rural and urban development plan. In comparison, the majority (46.0%) of the selected community expressed that CPEC is a project of all above-deliberated opinions. (Pakistan, 2017) surveyed in about CPEC, and this survey shows that the Pakistani community perceived that this project is significant for Pakistan’s development [49]. Toor, [50] examined that the CPEC will also develop Pakistan’s rural infrastructure, further improving Pakistan’s agriculture sector.

**Table 2:** Respondents’ knowledge about the CPEC project.

	Frequency	Percent
Yes	891	100.0
Electronic Media	217	24.4
Print Media	148	16.6
Both	407	45.7
Other	119	13.4
A road that links China and Pakistan	159	17.8
The most significant investment of China in Pakistan	164	18.4
Rural and urban development Plan	158	17.7
All of them	410	46.0
Economic Projects	159	17.8
Energy-related Projects	91	10.2
Infrastructure Development projects	114	12.8
Social Development Projects	386	43.3
All of them	141	15.8

Results also illustrate that (17.8%) of the respondents had an awareness that CPEC is an economic project, (10.2%) of them thought that it is an energy-related project, whereas (12.8%) of them were perceived that this project is an infrastructure development project. However, (43.3%) stated that CPEC is a social development project, and (15.8%) of the respondents thought that CPEC is a venture with all the above-discussed kinds of project. (Anonymous, 2016) CPEC is a multifaceted project, and these projects are also helpful for rural and urban development in Pakistan [51]. In the present study, roundabout (43.3%) of the respondents also perceived that this mega project is a social development project. (Observer, 2017) reported that under the CPEC project, many other projects also include infrastructure development, energy production, free trade zone, optical fibers, development of Gwadar port, established new technical colleges and hospitals, and all these projects uplifting the social development of Pakistan [52]. Due to the development of all these projects new job opportunities will be an increase, remote areas will be linked with big cities, technical and vocational institutes will also increase the social situation of the local peoples by providing training about the latest technology that will be shifted in the country Pakistan under the CPEC.

### 4.2 Multivariate analysis

#### 4.2.1 Convergent and discriminant validity

The current study determines discriminant and convergent validities through the values of average variance extracted (AVE) and composite reliability (CR), as shown in Table 3. According to [53,54], for convergent validity, the value of AVE should be greater than 0.50, and the value of CR must be greater than 0.60. However, for divergent validity, AVE’s square root’s value should be higher than the correlation value of that construct. The study results reveal excellent convergent and validities as all the values of CR are greater than 0.60, AVE is higher than 0.50, and the square root of AVE is also greater than the correlation values.

**Table 3:** Construct reliability and validity.

	Cronbach’s Alpha	CR	AVE	CA	ID	KS	RD	SD	TD
**CA**	0.912	0.938	0.791	**0.889**					
**ID**	0.879	0.903	0.538	0.187	**0.734**				
**KS**	0.907	0.926	0.642	0.1	0.315	**0.801**			
**RD**	0.922	0.939	0.719	0.244	0.307	0.168	**0.848**		
**SD**	0.936	0.946	0.662	0.22	0.573	0.509	0.499	**0.814**	
**TD**	0.925	0.939	0.658	0.308	0.528	0.351	0.678	0.659	**0.811**

Note: N = 891; Diagonal elements (in bold) are the square root of the AVE; CR = Composite reliability; AVE = Average variance extracted; CA = CPEC Adoptability; ID = Infrastructure Development; KS = Knowledge Sharing; RD = Rural Development; SD = Social Development; TD = Tourism Development.

#### 4.2.2 Measurement models

Hypotheses were tested using structural equation modeling (SEM) through SmartPLS V.3.2.9 package. SEM model was used to check the relation between different independent, mediation, and dependent variables. Anderson and Gerbing, [55] recommended that before hypotheses testing, the measurement model fitness should be tested first. To test the model fitness, confirmatory factor analysis (CFA) was conducted, and results are presented in Table 4. There are six latent variables in our dimension model. To test the measurement model fitness, we have used the most common fit indices such as Standardized-root-mean-square-residual (SRMR), Normed fir index (NFI), and Chi-Square(X2) values. SRMR value is a standardized-residuals index that is settled among perceived covariance and hypothesized matrices, which shows model fitness measurement [56]. Fit indices results shown good model fitness was achieved (SRMR = 0.062, Chi-Square = 5009.865, and NFI = 0.825). All these values are acceptable as a good model fit.

**Table 4:** Model fit.

	Estimated Model
SRMR	0.062
d_ULS	3.526
d_G	0.954
Chi-Square	5009.865
NFI	0.825

SRMR =; Standardized Root Mean Square Residual; d_ULS = Unweighted Least Squares Discrepancy; d_G = Discrepancy Geodesic; NFI = Normed Fit Index.

Moreover, standardized factor loadings for all items were greater than 0.60. Hence no item was dropped from the final analysis as shown in Table 5. According to [57,58], indicator loading below 0.40 should be removed from the model. Additionally, the multicollinearity of all items was measured by variation influence factor (VIF) value. The standard range of VIF is less than 10, and <5 considered as good. For this work, VIF values are less than <5, which means there is no multicollinearity problem among all influential constructs.

**Table 5:** Measurement of research model with factor loading and VIF values.

Constructs	Items	Factor Loadings	VIF
**CPEC Adoptability**	CA1 CA2 CA3 CA4	0.854 0.893 0.896 0.913	2.217 3.369 3.355 3.238
**Infrastructure Development**	ID1 ID2 ID3 ID4 ID5 ID6 ID7 ID8	0.708 0.693 0.671 0.715 0.769 0.776 0.771 0.758	2.124 2.015 1.923 2.078 2.049 2.156 2.014 2.040
**Social Development**	SD1 SD2 SD3 SD4 SD5 SD6 SD7 SD8 SD9	0.823 0.812 0.815 0.804 0.804 0.817 0.805 0.816 0.829	2.526 2.418 2.420 2.338 2.300 2.507 2.316 2.435 2.545
**Tourism Development**	TD1 TD2 TD3 TD4 TD5 TD6 TD7 TD8	0.765 0.872 0.853 0.864 0.807 0.739 0.806 0.772	2.088 3.818 3.692 3.724 2.724 1.992 2.774 2.288
**Rural Development**	RD1 RD2 RD3 RD4 RD5 RD6	0.815 0.889 0.867 0.868 0.862 0.785	2.232 3.779 3.477 2.869 2.748 2.088
**Knowledge Sharing**	KS1 KS2 KS3 KS4 KS5 KS6 KS7	0.807 0.804 0.797 0.795 0.814 0.799 0.793	2.155 2.182 2.081 2.087 2.217 2.118 2.038

### 4.3 Test of hypotheses

#### 4.3.1 Direct effect

Beta (β) value was calculated to observe the significance level of the proposed Hypothesis. Beta value explains the possible variation in the dependent factor from the independent factor. According to the Hypothesis research model, the Beta value for each relationship was calculated in Table 6. Additionally, the T-statistics technique was used to authenticate the significance of the beta value for each path. Hypotheses were tested using structural equation modeling (SEM) through SmartPLS, and results are shown in Table 6. Results reveal that infrastructural development positively and significantly associated with rural development (β = -0.13, p < .001), but the relationship between infrastructure development and knowledge sharing is insignificant (β = 0.03, p>.05). Results also demonstrate that social development positively influences rural development (β = 0.138, p < .001) and knowledge sharing (β = 0.479, p < .001). Moreover, tourism development also has a positive and significant impact on rural development (β = 0.646, p < .001), but an insignificant effect on knowledge sharing (β = 0.019, p>.05). The results also show the positive impact of rural development on CPEC adoptability (β = 0.233, p < .001). Furthermore, the results also reveal significant and positive effects of knowledge sharing and CPEC adoptability (β = 0.061, p < .001). All results are consistent with our predicted relationships; hence, all hypotheses were well supported and accepted instead of H1b and H3b.

**Table 6:** Test of hypothesis (Direct effect).

Hypothesis	Std.Beta (β)	T-Value	P-Value	Result
ID → RD	-0.113	3.553	0.000	Accepted
ID → KS	0.03	0.659	0.510	Rejected
SD → RD	0.138	3.424	0.001	Accepted
SD → KS	0.479	6.448	0.000	Accepted
TD → RD	0.646	13.386	0.000	Accepted
TD → KS	0.019	0.33	0.741	Rejected
RD → CA	0.233	6.642	0.000	Accepted
KS → CA	0.061	2.232	0.026	Accepted

Note: ***p < .001; ID = Infrastructure Development; SD = Social Development; TD = Tourism Development; RD = Rural Development; KS = Knowledge Sharing; CA = CPEC Adoptability.

#### 4.3.2 Indirect/Mediation effect

**Fig 3:**
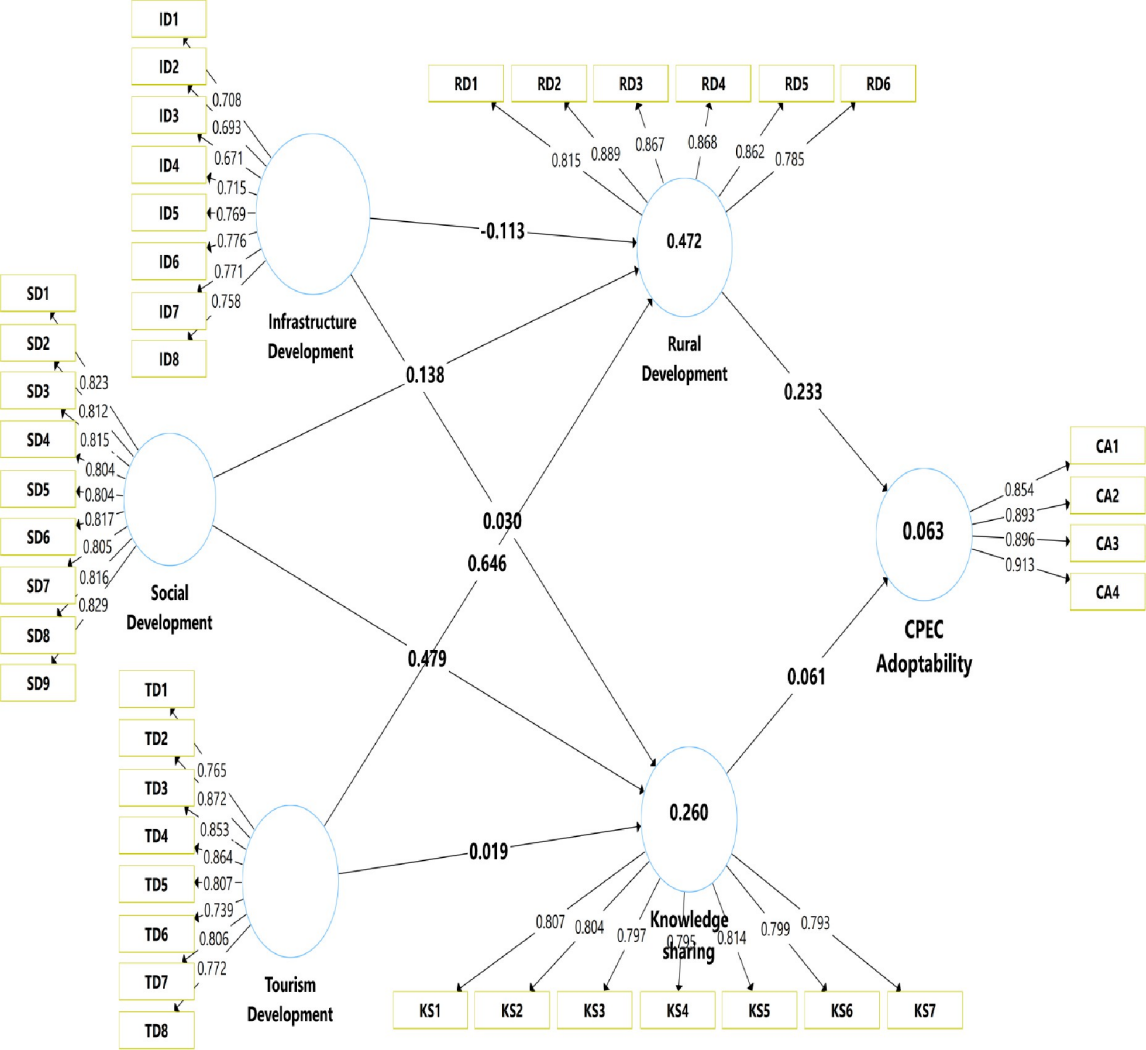
The SEM results with beta (β) values.

**Fig 4:**
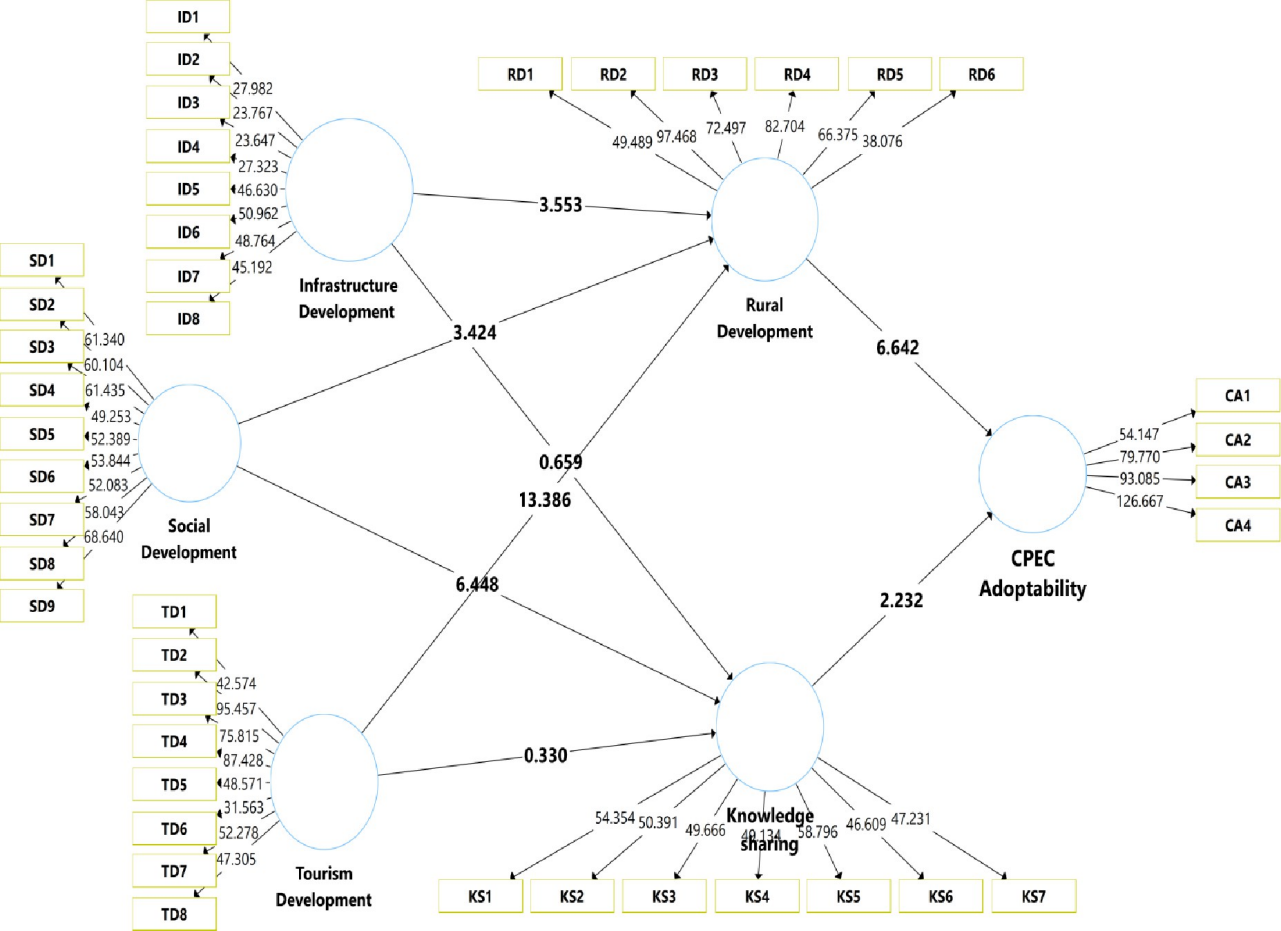
The SEM results with t values.

Results of indirect effects are shown in Table 7. The results show significant rural development mediation between infrastructural development and CPEC adoption (β = -0.026, p < .001). In contrast, there is no mediation of knowledge sharing found between the relationship of infrastructural development and CPEC adoptability (β = 0.02, p>.05), and tourism development and CPEC adoptability (β = 0.001, p>.05). Furthermore, the results show significant mediation of knowledge sharing between the relationship of social development and CPEC adoptability (β = 0.029, p < .001) and significant mediation of rural development between social development and CPEC adoptability (β = 0.032, p < .001), and tourism development and CPEC adoptability (β = 0.151, p < .001). These results are also presented in Figs 3 and 4.

**Table 7:** Test of hypothesis (Indirect/Mediation effect).

Hypothesis	Std. Beta (β)	T-Value	P-Value	Result
ID -> KS -> CA	0.002	0.541	0.589	Rejected
ID -> RD -> CA	-0.026	3.386	0.001	Accepted
SD -> KS -> CA	0.029	2.227	0.026	Accepted
SD -> RD -> CA	0.032	3.055	0.002	Accepted
TD -> KS -> CA	0.001	0.292	0.770	Rejected
TD -> RD -> CA	0.151	5.466	0.000	Accepted

Note: ***p < .001; Note: ***p < .001; ID = Infrastructure Development; SD = Social Development; TD = Tourism Development; RD = Rural Development; KS = Knowledge Sharing; CA = CPEC Adoptability.

## 5. Discussion, managerial & practical implications of the study

### 5.1 Discussion

The study’s primary theme is to examine the CPEC development for local communities’ perceptions regarding investment opportunities, education, health, and employment, which need to explore and consider the community-based benefits for Pakistan’s societal development. A research model for CPEC adoptability was proposed based on prior literature. This study data was collected in different provinces of Pakistan, such as Gilgit Baltistan, Khyber Pakhtunkhwa, Punjab, and Sindh. The mediation role of rural development and knowledge sharing was also observed. Several research papers are available on CPEC. Most of the studies emphasize the importance of CPEC with various aspects, challenges faced by implementing projects, social effects, and current developments in CPEC. However, recent research tries to emphasize the impact of CPEC adoptability on the public. The local people of Pakistan on different routes of CPEC within Pakistan will get many opportunities for employment and business, which further enhance their income level. Additionally, the necessary facilities of life such as health, education, transportation, market, and banking can be easily accessed through the CPEC in Pakistan’s remote areas. The victorious completion of the CPEC will help provide easy access to education and health facilities.

#### 5.1.1 Infrastructure development under rural development and knowledge sharing

This study explored the local neighborhood benefits and its connection with community support for CPEC. The results indicated that the transportation infrastructure network has a major impact on the local community of Pakistan. The governments of Pakistan and China, who are the partners of the CPEC project, have made huge investments in the project towards the transportation and infrastructural development. The infrastructural advancement will directly benefit the local population and indirectly help in poverty alleviation. Roads and transportation will create jobs, promote local economy, and raise living standards of the local communities. These findings are in line with the previous research conducted on road transport infrastructure. Hypothesis H1a of this study definite that Infrastructural Development positively associated with Rural Development. This study’s findings indicate the significant positive relationship between these variables, so our first hypothesis was accepted successfully. With infrastructure development, the local community benefits from improving health facilities and getting new business opportunities. This project also offers millions of new jobs. These factors will be restructuring the urban lifespan and play an active role in the progress of Pakistan. The findings of this study are consistent with the previous literature [4,9,12]. However, H1b is not supported because this study’s findings are not reliable with social exchange theory and previous literature [56,59]. Results of direct effects are shown in Table 6.

#### 5.1.2 Social development under rural development and knowledge sharing

Moreover, Social Development influence on Rural Development and knowledge sharing also show the positive relationship that indicated supporting H2a and H2b. Our study support [9,35], who demonstrates that CPEC helps achieve economic goals and helps achieve social goals that comprise education facilities, increase the quality of life, and provide job opportunity. This study also agrees with the study of [60,61] who indicate that there is a need to explore the perception of the local community by taking initiatives, such as basic necessity and need which will increase the quality of life, in this way positive change will come in residents’ social, personal and economic wellbeing. CPEC will influence household welfare and lives through industrial zones, competitiveness, and growth like SMEs, import, export, health, and education [62,63]. If the local Pakistani community stabilizes their desirable status, there will improve overall social conditions and balance life quality [4,24–26]. Menhas [4] reported that after the development of all these projects who started under the umbrella of CPEC (overcome the energy crises, infrastructural development, and established new business units) Pakistan ’s socio-economic condition will be improved automatically. Furthermore, this flagship project creates thousands of new job opportunities for local communities, rural areas connected with big cities, the new educational centers should be open, health facilities also improve, adopted new technologies all these factors also improve the social condition of Pakistan.

#### 5.1.3 Tourism development under rural development and knowledge sharing

This study also revealed that tourism development positively influences rural development H3a also supported. Likewise, because of the CPEC, the pristine tourist destinations with improved roads, power infrastructure, and pipelines will soon be fully accessible to tourists. Tourism is a lucrative industry and Pakistan is quite rich in tourist destinations. Therefore, the prospective influx of the tourists upon the project completion will highly boost national economic growth and specifically benefit the local communities. Moreover, tourism demand will also be enhanced due to the connectivity and approach and facilities in tourist resorts due to the development projects of CPEC [19,24,64]. Because of the country’s vast tourism potential, the tourism industry is mainly focused on attracting foreign tourists. Northern areas of Pakistan are known for tourist destinations and already attract huge influx of tourists mostly from Pakistan. With improved infrastructure and facilities, presence of foreign tourists will also be noticed soon. Likewise, the province of Baluchistan is blessed with breathtaking natural beauty and a diverse range of climates with huge potential to boost tourism. The CPEC project offers several opportunities for tourism investment from Khunjerab to Gwadar, and the Gwadar Port is specifically focused on tourism for potential revenue sources. The tourism industry contributed 5.9 percent to the GDP of Pakistan in 2019 (World Travel & Tourism Council), employing 3.9 million people. According to estimates, if the tourist industry of Pakistan grows on the similar pattern as that of China’s, the GDP contribution rate will increase to 11 percent, creating 2.37 million additional jobs.

Therefore, H3b tourism development positively influences on knowledge sharing is rejected; findings of this study are not consistent with and previous literature [65–67]. Some found a positive relationship between local community satisfaction and projects under CPEC associated with road and transport infrastructure development [68,69].

#### 5.1.4 Knowledge sharing as a mediator of multifaceted development

CPEC is an effort towards building an economic corridor for encouraging bilateral connections through infrastructural development; exploring mutual collaboration, business, trading, and transportation; and creating fast communication networks for regional connectivity to benefit both the stakeholder countries. CPEC aims to catalyze the development phenomenon in Pakistan by strengthening its economy via building mega transportation systems along with the subsidiary projects focused on energy needs of the country, and special economic zones as economic initiatives.

The knowledge about the prospective benefits of CPEC to Pakistan needs to be disseminated to the public and the local communities especially where CPEC projects are operational. This knowledge sharing will potentially offer more value to the project by updating the stakeholders on the advantages that it will reap for them in the near and far future. Information sharing on the project will positively mold the local communities’ perceptions about the project and is most likely to create a harmonious and welcoming attitude of the locals towards the project and the workforce involved. Most importantly, knowledge sharing is critical for achieving long-term goals and expected outcomes of the CPEC project. Therefore, it is pertinent to keep the communities updated on the mega project and its segments, and how the whole effort will benefit them in the long run. However, in the presence of knowledge sharing, both indirect effects (0.589 and 0.770) between infrastructural development, tourism development, and CPEC adoptability are insignificant. But in the reality of knowledge sharing, the indirect impact of social development (0.026) is positive and significant. Results of indirect effects are shown in Table 7.

#### 5.1.5 Rural development as a mediator of multifaceted development

Rural development plays a mediating role between infrastructure development, social development, tourism development, and CPEC adoptability [70]. Results also showed that there is a significant and positive link between Rural Development and CPEC Adoptability; hence H4 is accepted. There has been a rural/urban divide in Pakistan, partially due to the discrimination in development projects which are in general urban-centric in development policies. The primary routes of CPEC are mostly located in the rural areas, which has highlighted the symbolic significance of these so far neglected regions. CPEC development projects will improve the infrastructure of rural areas, creating a positive shift in the lives of the inhabitants.

Local communities will benefit from CPEC projects in a variety of ways, which include but are not limited to creation of a network of roads and transportation, promotion of tourism in these areas, and initiation of business ventures in the regions, which will bring development to these regions and improve living standards of the locals. Local residents and regions have already started noticing the positive effects on the healthcare facilities and employment prospects since the launch of CPEC projects. CPEC routes, in particular, will connect the rural areas, which were by far disconnected and deprived, with the mainstream routes, [15,71,72]. Results of direct effects are presented in Table 6.

The mediation model’s finding showed that all indirect effects (0.001,0.002 and 0.000) between infrastructural development, social development, tourism development, and CPEC adoptability are positive and significant in rural development. Moreover, this study [50] favors who analyzed that CPEC also focused on the development of rural infrastructure, improve the rural electricity and irrigation system, provide the batter agriculture seed, improve the agricultural crop mechanism, that will provide more easy access to the Pakistani farmer to sell your products all over the world and get good profit. Results of indirect effects are shown in Table 7.

### 5.2 Managerial & practical implication

The present study would help make valuable contributions to the existing literature related to CPEC and its positive impacts on local communities. This study is beneficial to almost all stakeholders. It has several theoretical and practical implications for Government, policymakers, and researchers as well. For theoretical implications, the present study contributes to social exchange theory that relates local community support to projects under CPEC. The survey results ensure that the local community knows that projects under the umbrella of CPEC are advantageous for them in all spheres. Results also show that CPEC will be beneficial in imparting knowledge to ordinary people. They would be familiar with new techniques, and in this way, they can show their efficiency and, in turn, increase productivity. Local communities can enjoy their high living standard by actively participating in socio-economic activities. This is possible only after getting knowledge and technical skills. However, projects under the umbrella of CPEC are still at the early stage of development. So, considerable research is needed. In the context of practical implications, the present study recommends to the officials of the CPEC in terms of local community perception. Because Pakistan’s considerable part of the population resides in local communities that need to be lifted socially and economically. Keeping in view the condition and opinions of local communities, policymakers would make policies and suggest that the Government allocate more funds or focus on constructing technical education centers and hospitals.

### 5.3 Findings for policy makers

Significant variations in the perception of the local populations in relation to the China-Pakistan Economic Corridor (CPEC) need to be considered especially by the officials involved in the local community segments of the project while simultaneously considering the consequences of the strategies utilized with respect to the perceptive variations. For better outcomes and smooth completion of the mega project and its subsidiaries, it is important to guarantee local communities’ economic and social participation in all segments of the CPEC project.

Economic initiatives can extend to the construction of infrastructural facilities such as small and medium-sized companies, restaurants, industries, and gas stations which would bring development to the area and create employment opportunities for the local people while enhancing their living standards. Moreover, business activity can be furthered in these areas through purchase of local products and involvement of locals in business activities.

Furthermore, promotional policies are also critical in this regard. Development practitioners need to formulate policies to generate more appealing opportunities for capital accumulation and business-friendly environment. Cable and media can be utilized for information dissemination and to create awareness on the outreach programs.

The Belt and Road Initiative (BRI) is expected to boost several investment activities, resulting in increased economic growth in both the countries. It is also anticipated that the project will lead to enhanced prolific initiatives such as travel, tourism, business, and trade. In addition, BRI is expected to afford more fellowship programs, research opportunities, and training facilities to students as well as education facilities for adults and children in the rural areas. Given the importance of education as an important factor of socioeconomic growth; and tourism, FDI, and infrastructural advancement as the tools of economic development, BRI benefits are quite lucrative and promising. That in view, the management and policymakers should devise policies that not only focus on the development of the local community but also go with the expectations of the local community to avoid any challenging circumstances during and after the completion of the project.

## 6. Conclusions

For several years, the world has changed a lot, from political warfare to economic welfare. Research and development by China have mystified the world for its performance in all sectors. China showed outstanding performance, especially on economic grounds, with the latest industrial techniques that made China a superpower at the international level. Luckily, China is Pakistan’s neighbor and also an all-weather friend of Pakistan. Pakistan always enjoys the moral support of China. The climax of this friendly relation led towards CPEC. Pakistan is one of the dominant countries to initiate the OBOR project with China that promises long-term benefits. These benefits include knowledge sharing, rural areas development, tourism development, socio-economic development, and infrastructure development. This project would be fruitful, especially for local communities. Collection of projects under CPEC would help improve local communities’ lifestyles by providing technology, employment opportunities, etc. This study examines the social, infrastructural, and tourism effects of CPEC through the mediation role of rural development, knowledge sharing, and CPEC adoptability for a local community in Pakistan. To estimate the results, univariate and SEM analyses were conducted. The current study concluded that CPEC plays a vital role in Pakistan’s social, infrastructural, and infrastructural development. It is also expected that this project would positively impact the rural of Pakistan. Similarly, our study also concludes that CPEC can bring positive infrastructure, rural development, energy, and social development projects in Pakistan. With the improvement in industrial parks and free economic zones, it is also observed that it would be beneficial to combat poverty and bring prosperity to Pakistani societies. This study has investigated the perception of resident’s adoptability in Pakistan. Simultaneously, the same perception may also be observed with the local Chinese community. Comparative analysis between both countries of residents’ benefits about the CPEC development projects needs to be investigated further. Most of the positive effects of the CPEC development projects have been examined on the living standards. In contrast, adverse effects related to the CPEC project and other issues like securities issues may also be examined in further studies. Organizations run by the government and private sector and business community require to play a crucial role in making CPEC projects fruitful.
